# Hardware–Software Partitioning for Real-Time Object Detection Using Dynamic Parameter Optimization

**DOI:** 10.3390/s23104894

**Published:** 2023-05-19

**Authors:** Corneliu Zaharia, Vlad Popescu, Florin Sandu

**Affiliations:** Department of Electronics and Computers, Transilvania University, Bdul Eroilor 29, 500068 Brașov, Romania; corneliuzaharia@unitbv.ro (C.Z.); sandu@unitbv.ro (F.S.)

**Keywords:** computer vision, artificial intelligence, hardware accelerators, object detection, hybrid implementations, adaptive hardware resources integration, FPGA, embedded systems, memory bandwidth

## Abstract

Computer vision algorithms implementations, especially for real-time applications, are present in a variety of devices that we are currently using (from smartphones or automotive applications to monitoring/security applications) and pose specific challenges, memory bandwidth or energy consumption (e.g., for mobility) being the most notable ones. This paper aims at providing a solution to improve the overall quality of real-time object detection computer vision algorithms using a hybrid hardware–software implementation. To this end, we explore the methods for a proper allocation of algorithm components towards hardware (as IP Cores) and the interfacing between hardware and software. Addressing specific design constraints, the relationship between the above components allows embedded artificial intelligence to select the operating hardware blocks (IP cores)—in the configuration phase—and to dynamically change the parameters of the aggregated hardware resources—in the instantiation phase, similar to the concretization of a class into a software object. The conclusions show the benefits of using hybrid hardware–software implementations, as well as major gains from using IP Cores, managed by artificial intelligence, for an object detection use-case, implemented on a FPGA demonstrator built around a Xilinx Zynq-7000 SoC Mini-ITX sub-system.

## 1. Introduction

Most of the Computer Vision (CV) solutions are now driven by Artificial Intelligence (AI). With Real-Time (RT) applications, the implementation of the computer vision algorithms becomes challenging due to the numerous calculations involved and the large amount of data to be manipulated in a restricted span of time.

For this reason, the Typical Software (SW) implementation of the computer vision algorithms is no longer acceptable, since a number of Key-Point Indicators (KPIs) specific to RT operations cannot be met effectively. When new image processing algorithms are developed in a powerful computational environment, the metrics are related to the algorithm quality (F1 scores, detection rates, precision–recall curves, etc.—as detailed in Appendix A). In the case of real-time computer vision, there is a specific requirement to fit in the available budgets for execution time, memory bandwidth, or power consumption (budgets that are more critical for mobile applications). Therefore, all these KPIs must be balanced in order to provide efficient solutions.

A wide range of hardware (HW) accelerators for artificial intelligence algorithms is available nowadays, solving specific needs, such as the need for increased computational capacity and reduced energy consumption—to mention only the most prominent ones.

The optimization of the computer vision algorithms should start from the proper understanding of the KPIs and balancing them adequately.

Noteworthy is the classical KPI of Integrated Circuits (IC) development—the PPA (Performance–Power–Area) category, such as processing capacity, energy consumption, and silicon area. Major KPIs in real-time Image Processing are the quality of algorithms and the two KPIs addressed by this paper, efficiency of memory use and the flexibility in use. Last, but not least, for the hardware accelerators in RT CV applications, privacy by design and privacy by definition are carefully addressed, with KPIs, such as privacy and security, or utilization risks (such risks regard not only data exposure, but also unwanted exposure of the algorithms themselves).

The hardware accelerators optimization may take advantage of the artificial intelligence algorithm specificity, especially in the case of scarcity of available bandwidth or available energy. In this context, we introduce the idea of adaptively integrating the hardware resources based on the decisions performed by an artificial intelligence algorithm. This approach improves the merit factor of a defined solution—a factor that can be computed as a weighted sum of all KPI metrics (with critical and even disqualifying KPIs).

The opportunity for our approach is based on the fact that, nowadays, the silicon gates cost is significantly reduced, so we can use only the needed hardware IP Cores (Intellectual Property Cores) at a defined time, while the others are kept latent (and consequently consume less or no energy). Another opportunity in our approach to critical RT and CV problems comes from the fast interaction between software and hardware parts of the algorithms running in modern embedded systems.

Our goal was to implement a mechanism, which uses artificial intelligence (SW-driven), firstly, for adaptive configuration management by hardware block selection and aggregation and, secondly, by computing the parameters needed to instantiate these hardware resources. Due to the predictive nature of the artificial intelligence computations, the dynamic change of best parameters setup for the hardware blocks also ensures an optimal processing of near-future data in hardware. The motivation of our research is to address the ecosystem of data–algorithms–HW–SW in order to operationalize AI-driven optimizations for RT CV applications. Each of the four parts of the ecosystem affects and is affected by the other ones, in an evolutionary relationship, so each part has to be adaptive in order to bring an optimized ”behavioral” contribution to the overall system performance. This requires a complex co-design, including data structuring and segmentation, as well as algorithm hybrid partitioning.

The objectives of the presented paper are the conceptualization and validation of the artificial intelligence potential in managing hybrid hardware–software solutions (parts of the main RT CV algorithm are implemented in hardware), on the one hand, and the practical implementation of the computer vision solution into an FPGA (Field-Programmable Gate Array) demonstrator, on the other hand.

Regarding the apparatus, the implementation of computer vision solutions was initially done solely in software, using the C and C++ languages, then software profiling was applied to understand the areas appropriate for hardware acceleration. The Artificial Neural Networks (ANN) algorithms used started from public implementations using typical NN frameworks (like Tensorflow or Pytorch) and then optimized for embedded software implementation (in C++). The implementation of the hardware accelerators was carried out using Verilog or SystemVerilog description of the IP Cores and logical synthesis, place and route, static timing analysis, and sign-off using industry-standard tools for FPGA (Xilinx toolchain). Verification for the IP cores was performed using Universal Verification Methodology (UVM) and SystemVerilog.

The novelty of the proposed system resides in the dynamically reconfigurable hardware accelerators that process image frames in real-time. An actual frame can be adaptively processed using image primitives generated from the previous frame and stored efficiently, with the advantage of better memory bandwidth utilization.

The paper is organized as follows: after the introduction in Section 1, the Section 2 presents the related work in the area. Section 3 approaches the proposed hybrid hardware–software solution, as well as the hardware accelerator sub-system and its anticipative dynamic hardware parametrization by the artificial intelligence. The results of the implementation are presented in Section 4, and they are validated on a FPGA demonstrator. Finally, in Section 5, conclusions are drawn.

## 2. Related Work

Different types of computational architectures are used to implement artificial intelligence algorithms [1].

Standard computational architectures are based on general-purpose processors (e.g., Central Processing Units—CPUs) and parallel processors (e.g., Digital Signal Processors—DSPs, Graphical/Vectorial/Neural/Accelerated-Processing Units—GPU/VPU/NPU/APU).

Focusing on related work on mobile solutions for Edge artificial intelligence, among the most relevant, one can mention the CPUs from Intel (Atom) [2], ARM (Cortex) [3], Beyond (BA22) [4], and the ”Reduced Instruction Set Computer” open architecture RISC-V [5]. Although suited for very flexible implementation of artificial intelligence algorithms and having the lowest risk in use, standard CPUs are not optimized for artificial intelligence because of their lower computing capacity and high power consumption.

Parallel processors still have standard architectures and are not explicitly intended for artificial intelligence applications, but they perform much better in this domain as compared to classic processors. Most of these architectures use Very Long Instruction Words (VLIW), consisting of Single Instruction Multiple Data (SIMD), an important advantage for the implementation of artificial intelligence algorithms, which require a large number of repetitions of the same operations for distinct data [6]. Risks in use remain low, but, in comparison with CPUs, flexibility in use decreases a little due to specific resource programming requirements. The biggest disadvantage of these computational architectures lies in the high energy consumption. Additionally, the occupied area on silicon is a drawback, which leads to higher costs of these architectures.

Among the most notable parallel processors, one can mention Qualcomm Hexagon DSP [7], Texas Instruments EVE (Embedded Vision Engine) DSP [8], CEVA DSP [9], nVidia GPU [10], ARM Mali GPU [11], Imagination PowerVR GPU [12], Movidius VPU [13], Samsung NPU [14], AMD APU [15], as well as Verisilicon VIP (Vision Image Processor) [16].

Another class of computational architectures includes the more specialized Application-Specific ICs (ASIC), and, here, we must include the FPGAs [17]. Among the FPGAs most suitable for the implementation of artificial intelligence algorithms are those integrated into the Xilinx SoC (Systems-on-Chip) of the Zynq family [18]. A similar solution, from Intel, is Arria and its cheaper alternative, Cyclone [19] (both formerly from Altera). These architectures include sub-systems with fixed functionality (control units, processors, peripherals), as well as programmable logic that can be easily used for artificial intelligence algorithms. Specific to these artificial intelligence solutions is the implementation of DSP sub-systems inside the programmable logic [20], which contributes to better performance. The advantage of these architectures is the flexibility in use and the low implementation risk due to the option for completely reprogramming the device structure (down to the bit level). A major disadvantage is the energy consumption, quite large as compared to the above-mentioned architectures, so FPGAs are more suited for demonstrators (such as in our proposed solution) or a limited number of special appliances.

The last class of architectures depicted by the literature includes those implemented in specialized ICs. Noteworthy are Google’s Tensor Processing Units (TPU) chips [21], IBM’s TrueNorth [22], and Intel’s LakeCrest/Sprint Crest/Spring Hill [23]. All these offer a high computing capacity, being highly specialized for the implementation of artificial intelligence algorithms. Memory access (both local and external) is optimal in relation to the other above-mentioned architectures. Energy consumption is another major advantage of these architectures, due to the specific optimizations for the implemented algorithms. However, the area occupied by silicon is large, and the cost of these devices can be very high, especially if the production volumes are not extremely large. In addition to the cost, the lack of flexibility in use is another disadvantage of these ICs, and modifying their architecture or using it in ways that were not decided in advance being very unlikely. The risk is high, both in terms of the design of the devices and the algorithms used. From the analysis of the related work, it turns out that there are no clear winner architectures in terms of specific criteria and KPIs, each architectural model having its pros and cons. That is why hybrid or heterogeneous computational architectures deserve special attention—they will be addressed in our paper, with the belief that they can find balance between the advantages and limitations of the above-mentioned architectures, having a great potential for RT CV driven by artificial intelligence.

Novel architectures of the imaging pipeline for CV are envisioned as reconfigurable, balanced by a system-level trade-off between accuracy and computational cost [24,25]. Accelerators with low hardware overhead—due to module reusing techniques in the pipeline—achieve fast matching of visual templates, as well as high energy efficiency [26].

Memory issues were specially addressed by researchers, up to the crossbar for synapses [27], in cores for neuromorphic computing [28]. Hardware accelerators for artificial intelligence, with state-of-the-art of GPU/FPGA/ASIC solutions, are presented in [29], ranging from processing-in-memory capabilities from SRAM/DRAM (Static-/Dynamic- Random Access Memory) to the emerging memories (memristor/spintronic).

FPGA-based feature extraction in computer vision experimented hardware solutions that improve speed and resource utilization, with the decrease of digital signal processors (DSPs) used in the FPGA, moving some calculations to logarithms (and replacing multiplications with subtractions) [30].

Real-time object detection FPGA-based solutions have already been demonstrated in the literature. To reduce the computational complexity, these modern solutions can use a “non-neural network” (“non-NN”) approach, for instance, computing HOG (Histograms of Oriented Gradients [30]) with Support Vector Machines—SVM [31]. If using a neural network, such as the public reference YOLO (“You Only Look Once”), different solutions aimed to reduce the complexity of the YOLO-like NN to obtain results as close as possible to the reference network [32,33,34,35,36]. For this second approach, the cost of achieving real-time operation is a certain decrease of the algorithm quality.

The problem of resolution in the domain of image processing is approached by AI-based improvement of the quality (image restoration) [37]. Feature engineering and model estimation are important perspectives of AutoML (Automated Machine Learning) in computer vision [38,39].

Domain invariant representation is valuable for improving object detection algorithm quality [40,41]. To take advantage of the real-time operation, estimation of parameters to be programmed in the hardware blocks should be oriented toward extracting instance invariant features by disentangling domain-invariant features from the domain-specific ones [42].

## 3. Proposed Method

In computer vision, increased complexity image processing algorithms match the newest sensors performance (e.g., 48 MP resolution for image acquisition and 8 K resolution at 120 fps for video capture) in more frequent multi-camera and/or multi-optics systems that require a higher processing capacity and a greater memory bandwidth.

### 3.1. Rationale of a Hybrid HW–SW Solution for RT CV

**Figure 1:**
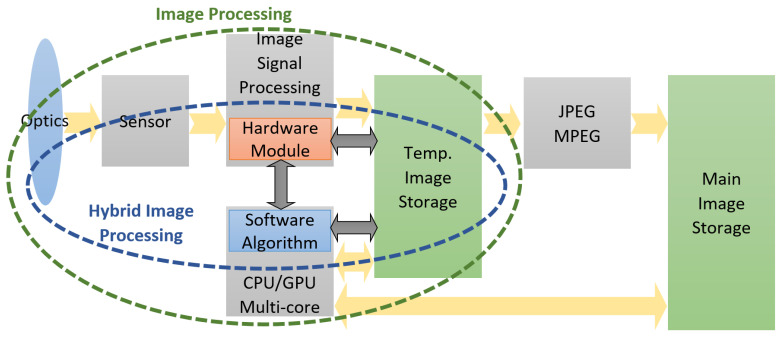
Hybrid HW–SW Image Processing Solution.

In order to address the challenges of memory bandwidth and energy consumption in mobile systems with computational imaging, we have proposed the hybrid approach presented in Figure 1.

This approach is based on the image processing algorithm partition into a hardware part (hardware modules integrated into the Image Signal Processing subsystem, ISP, for rapid and efficient access to images) and into a software part that—taking over the results from the hardware modules—finalizes the overall algorithm computations.

Thus, complex image processing algorithms no longer have a pure software implementation in which the computing system (with CPU/GPU processors) is solely in charge of running them.

An important share of the processing tasks (of lower complexity, but important in volume and speed) are moved to dedicated hardware components, which are controlled (and configured) by the software part.

Interfacing becomes very important for the close coupling of this hardware module with the other components of the data processing flow, so as to maximize the throughput needed for real-time operation. The temporary memory is mainly used to store the final results of the hardware processing, while the intermediate results are kept in the internal memory of the hardware modules. From the point of view of the software algorithm part, the hardware algorithm part is an accelerator for specific functions, configured according to the needs of the software part.

The advantages of this solution are given by a lower computing power required for the software algorithm (compared to the alternative of all processing done in software), significantly low memory bandwidth, and lower energy consumption. The compromise resides in a reduced flexibility in use. The benefit of the interfacing solution on memory bandwidth comes from the direct data accessing by the hardware part of the algorithms thus partitioned—from inside the ISP system. The significant advantage is the fact that it is not necessary to read the image (to be processed) again from the memory. The results of the hardware part of the algorithm can be transmitted directly to the software part, using methods, such as “mail-box” or communication through registers, or they can be stored in temporary memory as meta-data. Regarding the lower energy consumption, the same operations executed in the hardware module consume less power than using generic CPU or GPU resources. Depending on the performed operations and on the way in which they are intended to be optimized, our analysis has shown a power consumption of 10 to 100 times lower if dedicated hardware resources are used, as compared to the full software alternative.

The second challenge is given by the continuous increase in the size of the images to be processed, in parallel with the number of frames processed per second, which leads to a very large memory bandwidth needed by the image processing system.

The image processing system contains both the image signal processing module (ISP) and the general-purpose processing sub-system composed of the CPU and/or GPU. Data are stored and reused from the temporary memory (SDRAM). This approach assumes that at least one write and read from memory is required for each frame, an operation that sometimes exceeds the bandwidth provided by the external memory. The cost of an IC is directly proportional to the area of silicon consumed. For real-time image processing systems, most of the silicon area is occupied by the ISP, followed by the computing system (CPU/GPU). The reason why ISP traditionally consumes a significant area is given by its need to implement numerous buffers to retain the necessary image processing intermediate data. Considering the internal memories of the ISP, but also the cache of the general-purpose computing system, it can be concluded that the image processing ICs are memory-centric, with over 50% of the silicon area being consumed with different memory blocks.

### 3.2. The Anticipative HW Parametrization by the AI

**Figure 2:**
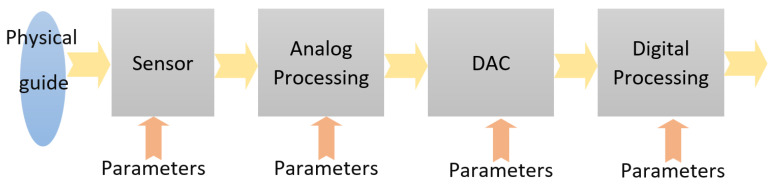
Real-time Image Processing Parametrization.

For RT CV systems, the typical image processing stages (shown in Figure 2) provide functions, such as signal reception, conversion of the signal to be acquired into an electrical signal (at the sensor level), signal processing in the analog domain, aAnalog-to-Digital Conversion (ADC) of the signal, and signal processing in the digital domain. Symmetrically, there can be considered further DAC (Digital-to-Analog Conversion), amplifying, actions (of the actuators), and so on. DSP is typically divided into generic processing of the digital signal and auxiliary rejection of noise and other distortion, normalization, etc. The data flow can be linear (each block uses the signal generated by the previous block, as it is) or nonlinear (in which the signal is stored and used by the next block in a specific way—different from the one generated by the previous block). Storage can be local (memories between two consecutive blocks of data flow), divided between several blocks (shared), or global.

In signal processing systems, each block of the data pipeline can be parameterized independently, depending on how it is used, having an output (out) as a function of the input (in) and of the parameters (param). In the following various situations, the expression ”a function of” was denoted, only symbolically, with f, so


out = f (in, param)
(1)

In simple processing blocks, parameters are constant:


param = K
(2)

Artificial intelligence can be introduced in the image processing pipeline, performing own specific pre-processing, proper artificial intelligence calculations, and post-processing. In our proposed solution, the special goal is a dynamic change of parameters based on the statistics on previously received signals:

param = f (statistics)
(3)

with

statistics = f (in)
(4)

This can lead to the adaptative behavior of RT CV blocks, for a better response to the input signal, e.g., detection of maximum signal level and automatic gain control (AGC) or adjusting the brightness of images (depending on the pixel brightness histogram). As for the dynamic feature of parameterization—needed for real-time operation—the main problem is the sudden change in the input signal. In this case, a too-long delay of the loop (input statistics parameterization) affects the global transfer function of the image processing system. Using artificial intelligence algorithms, changes in the input signal can be anticipated, and thus a predictive parameterization of processing blocks can be performed:


param = f (expected input)
(5)

This is one of the reasons for approaching the overall signal processing, such as the above-mentioned ecosystem.

Our proposed HW accelerator for computer vision, called AHIP (“Advanced Hardware for Image Processing”), processes, in hardware, the input data received from the video sensor, the results being subsequently used by higher-level image processing algorithms implemented in software. The AHIP includes a set of hardware modules that implement different image processing primitives, managed by a control sub-system with artificial intelligence. Segmentation of the data ‘of interest’, on different criteria, as decided by the artificial intelligence, consists of identifying sub-sets (‘regions’, ‘areas’–‘collections’) and extracting them to be treated in the dedicated hardware modules.

**Figure 3:**
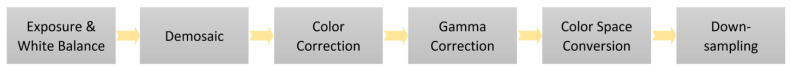
Typical Image Processing Pipeline (IPP) Flow.

After being acquired by the sensor, the image is processed (before its display or storage) in the Image Processing Pipeline (IPP), illustrated in Figure 3. It includes an exposure and white-balance module (1), a “de-mosaic” block (removing the mosaic effect) (2), a color correction block (3), a gamma correction block (4), a color conversion block (5), and a sub-sampling module (6).

When implementing a real-time video processing system, there are often specific constraints on the IPP, since image data is usually read from the memory for each stage of the IPP and then written back to the memory after each bunch of operations. Higher video resolution brings a significant challenge to memory bandwidth. To meet these challenges, our option was to implement the IPP elements of Figure 3 directly in dedicated hardware, for RT CV. The advantage is that IPP elements avoid, in this way, the constraint to write image data to memory after each processing step and to read back these data for each subsequent IPP operation. The downside is that the methods applied at each stage of the IPP could be less adaptable, with the entire IPP chain being already configured before entering the data from an image frame. Digital Still Cameras (DSC) implement a more sophisticated analysis of the image and scene compared with what a basic IPP can provide (as illustrated by the blocks in Figure 3). Image acquisition devices can now detect and track faces regions in an image scene; further on, they can analyze and detect imperfections in such regions, correcting such defects “on the fly” (in real-time). Imperfections, such as those caused by dust stains (on the lens), can also be detected to improve the quality of these regions. Image blur and/or image motion (translational and/or rotational) can also be determined and compensated. Recognized facial regions can then be associated with known people.

All these techniques are based on the analysis of an image scene. Usually, this involves reading blocks of image data from a memory, followed by different stages of processing these data. Intermediate data structures can be temporarily stored in the image memory for the needs of each scene analysis algorithm. In some cases, these data are specific to a single algorithm, while in others, data structures may persist in several different scene analysis algorithms.

In these cases, the image data need to be moved between the image memory and the image processor. If several algorithms are applied, the image data are usually read several times to perform the different image and scene processing operations on each image.

For most of the techniques mentioned above, the analysis may involve image pre-viewing, using also a relatively low-resolution stream captured by most digital cameras and used traditionally to provide an image in real-time on the camera screen. Thus, to correctly analyze the main scene, it is useful to secure at least two available images of this scene. If one or more preview images are stored, they are usually read more times, in combination with the main (full-resolution) image. In addition, processing may involve the temporary storage of under-sampled copies of pre-view images or under-sampled copies of the main image, produced to facilitate various scene analysis algorithms. In digital cameras, images are usually acquired individually, and it usually takes a substantial amount of time, even in the order of seconds, between image acquisitions for scene analysis and post-processing of individual images. Even if several images are acquired one after the other, for example, in the “burst” mode of a professional DSC, only a finite number of such images can be processed because of the limited memory. In addition, the processing of these images cannot be performed during the burst acquisition, but only after this is completed more sophisticated scene-based processing can be performed. Within a modern video camera, data are often processed at a frame rate of 30 fps or even more; due to memory constraints, data can be digitally compressed and immediately written in a long-term memory. Additionally, a low-resolution pre-view stream is typically not available (as in the case of a DSC), so manipulating a high-resolution video stream requires a significant memory bandwidth.

**Figure 4:**
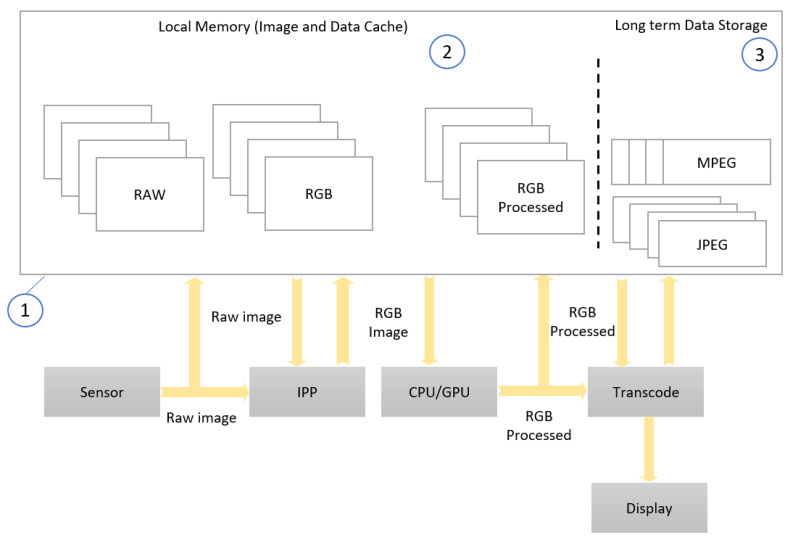
Typical Video Processing System Data Flow.

There are several important challenges to achieve in a high-resolution video acquisition device with regards to the benefits of the modern scene analysis techniques that are currently available in DSC. First, it is difficult to store and perform complex analysis of the scene on high-resolution images in the time that is available between video frame acquisitions. This is not simply a matter of CPU power, but, more importantly, a matter of bandwidth of the internal data transfer. The size of high-resolution images makes it difficult to circulate such an image “as it is” through the IPP and then towards a video compression unit before long-term storage. While some limited scene analyses may become possible through the above-mentioned hardware additions to the IPP, this would require even more rigid configurations and settings (of parameters), fixed before starting the acquisition in real-time of the video stream, so that these analyses cannot be dynamically adaptable—“responsive” to the ongoing scene. Secondly, image processing primitives cannot be shared by several algorithms without introducing very large buffers distributed in the IPP. This would lead to unreasonable hardware design requirements that would just replicate the currently existing solutions in a single IC, as shown in Figure 4.

Figure 4 illustrates conventional hardware to implement a typical processing for computer vision, with the other high-level functions in software.

For practical reasons, such a conventional hardware cannot support an optimal image processing mechanism. An alternative is to have separate hardware implementations for each scene analysis algorithm, but this will also lead to excessively constructive complexity, since each algorithm would use as a buffer a complete frame of the image in order to perform a complete analysis of the scene. While there are several implementation options for each component, we propose an original, AI-based solution that allows for a more comprehensive approach to scene analysis and image enhancement, applicable to RT CV systems.

The proposed hardware system—designed, implemented, and validated on a FPGA demonstrator—generates, in real-time a set of image primitives for each image received. Images are fetched serially, pixel by pixel, with much lower latency compared with the acquisition of a complete frame. The primitives are available immediately after the input image frame has been acquired and can be used to further process this current image frame simultaneously with the acquisition of the next image frame—pipelining. In addition, the meta-data obtained by processing the previous frame (in a frame sequence) can be used in conjunction with the current frame primitives. This allows for detailed frame-by-frame processing without the need to separately capture a stream of low-resolution pre-visualization video frames. This way, the real-time processing is anticipative (from the analysis of the previous frame, the content of the current frame is anticipated). The generation of different primitives that can provide such useful information for the current frame involves operations that are performed at the speed of 1 pixel per clock (CLK).

A series of different types of these image processing primitives are generated, providing useful information about the previous frame. Each type of primitive is generated by a processing chain that includes one or more blocks of pixel operations. These blocks are connected by several internal connections, which can be changed dynamically. In less complex cases, data connections may be fixed, but the primary input of the hardware subsystem can be switched between the sensor, the IPP, and the internal memory. At the same time, multiple blocks can share the same data entry. Furthermore, the outputs of several blocks can be combined using logical functions. The individual output of multiple processing blocks is combined into a single data word before being written to the external memory (SDRAM) to optimize memory usage and external memory bus usage. Due to differences in processing time in the different blocks, sync blocks are integrated with the circuit logic to correctly align the output data flows.

The generated image primitives can be used to significantly simplify the implementation of a set of accelerated image processing operations. In addition to object detection, face recognition, face beautification, red eye correction, and panorama stitches can be performed. This reduces the need to read and write the entire image frame from/to the external memory by the CPU or GPU. Most of the time, the relevant primitives and the current frame are read only once to analyze or improve the input image using a particular algorithm. The primitives for multiple algorithms can be read together only once—thus, all these algorithms can be executed for a single reading of the input image. This mechanism significantly reduces the memory bandwidth for processing a video stream. If separate read and write buses are available, an image frame can be processed by the software sub-system (at CPU/GPU level), while the next frame is acquired and pre-processed by the AHIP module.

In addition, this hybrid configuration allows data produced from the analysis of an image frame processed by the CPU/GPU to be returned (“fed-back”) to IPP or to the AHIP module to adapt the pre-processing of the next image frame. This detailed adaptation of both the overall image processing by IPP and the scene-specific image processing by AHIP allows for better performance and lower latency. This further enables the video acquisition to be adapted more quickly (in situations where, for instance, the lighting conditions are changing), based on an analysis of the regions of the object that is the subject of detection, as well as on an analysis of the color maps associated with that specific object. For this purpose, a frame counter (and the associated logic) can be used. At the end of each frame processing cycle, it is possible to reconfigure internal pixel processing chains. This may involve updating the LUTs (Look-Up Tables) content, changing the parameters of individual pixel processing blocks, or in some cases, reconfiguring the order or logical combination of blocks in a processing chain. Blocks are either selected or bypassed (even shut down, for important energy saving—On/Off). In more complex scenarios, data processing modules share an I/O (Input/Output) port on one or more internal data buses. Dual-port memories can be used to ensure almost simultaneous read/write operations.

**Figure 5:**
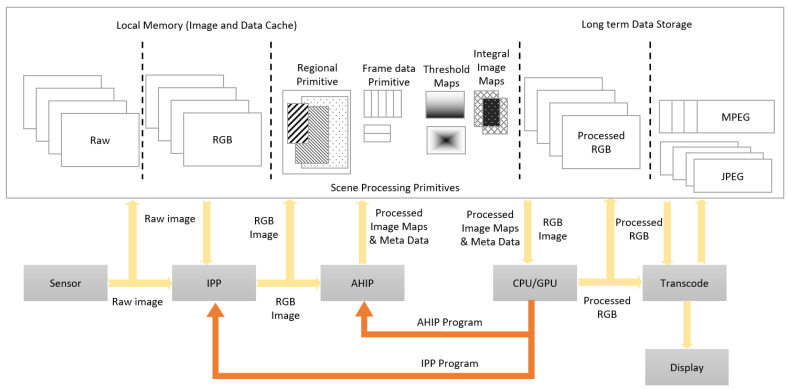
Dynamic configuration for real-time object detection system.

Compared with Figure 4 (which illustrates conventional hardware to implement an IPP), Figure 5 presents a hardware IPP with AHIP that takes over a direct data stream.

**Figure 6:**
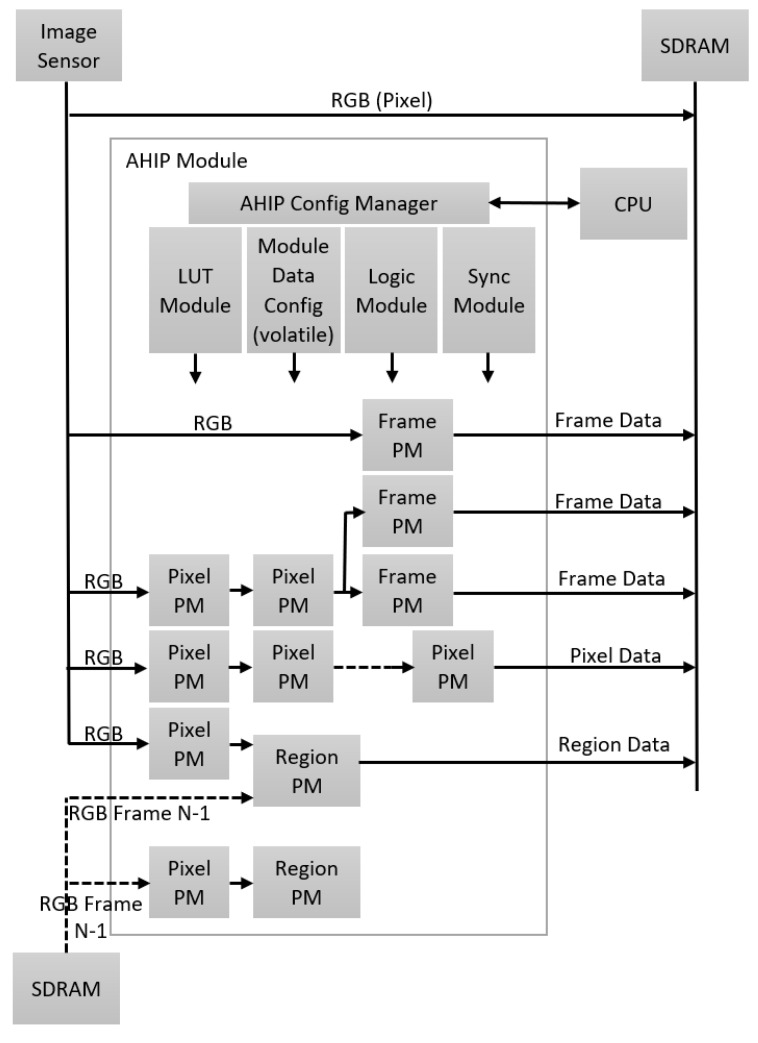
AHIP Block Using Generic Processing Modules.

Figure 6 schematically illustrates the AHIP module with several generic blocks arranged in processing chains for different image processing primitives. An image sensor, a SDRAM memory, the AHIP module itself, and a CPU are shown. The AHIP module includes an AHIP configuration manager that communicates with the CPU. The AHIP module also includes a LUT module, a data configuration module, a logical module, and a synchronization module.

As illustrated earlier in Figure 4, a part of RGB data is immediately stored in memory. Other RGB data are processed by the AHIP block through one or more pixel processing modules (PMs), one or more frame PMs, one or more region PMs, and one or more core PMs—for “kernels” (small square groups of pixels).

Certain RGB data can be processed in a frame processing module, and the frame data are stored in memory. The RGB data of an adjacent frame, such as a previous frame (numbered N−1), can be processed together with the RGB data, in a region processing block, as mentioned above. For instance, using consecutive frame data may help to detect regions where motion happens. The current frame and the previous one are compared, and areas, where a number of pixels are changed, represent the region of motion in the picture. The data processed by this region processing module can then be stored in memory as region data. It may often be appropriate to apply an algorithm to portions of an image—for example, to fully confirm the exact location of an object. These predicted regions of the object can be determined from the previous frames of the image and can be accompanied by a history of the movement of the object and of the camera on a series of previous frames. In this regard, dX and dY movements from frame to frame can be determined by the AHIP module and may be available with a short delay, after processing the last pixel of a frame. Similarly, the location and size of a region of the object can be accurately known for a number of previous frames of an object tracking algorithm, and both datasets may be available very soon after the processing of a new image frame has begun in AHIP. This data usefully allows for an accurate and dynamic estimation of the regions of the objects predicted for the current image frame.

Object regions, or regions of parts of objects, can initially be saved to a local memory. Usually, because memory is relatively expensive in a hardware module, there may be a limited number of “local object memories”, and they can be optimized—to minimize memory size. Indeed, in some implementations, such memories may be external to AHIP and may require bandwidth—to write these regions to the main external memory. In other implementations, the locations of these regions in the main image can be recorded, so that they can be accessed later in the image in the main memory. In a representative use case, such regions are stored internally within the AHIP module. However, since the size of the AHIP local memory may be too small for high-resolution video storage, these regions of the object can be stored together with the image in the main memory and reloaded to an AHIP secondary module while the next frame of the image is processed and written to the main memory.

The AHIP blocks that can be used, according to the decision of AI, are: Color Map, Color Space Conversion, Thresholding, Logic Functions (on these spaces), Histogram, Cumulative Histogram, Integral Image, Integral Square Image, Fixed Resampling, Complex Resampling, Profiling, and Offset. This is the intelligent configuration phase.

For example, when the image has a low contrast, the cumulative histogram block is needed to correct the contrast curve of the Region Of Interest—ROI. When the image is properly exposed, the cumulative histogram block is not used, so it is simply powered down.

### 3.3. Implementing the HW–SW Partition Using AHIP

**Figure 7:**
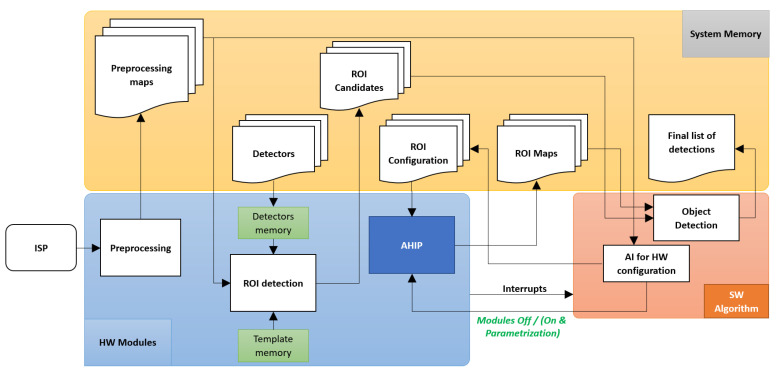
HW–SW interface for the Object Detection Algorithm.

In order to meet the requirements set out above, we have performed the partitioning of hardware–software so that the algorithm components that require extensive computing resources (according to the analysis of the algorithm profile) are implemented in the hardware, and the components that require intensive computing and, more important, that are running the complicated control tasks, are implemented in software. For this, the module that performs image pre-processing (PRE), the engine for ROI detection, and the ROI processing module (AHIP) were implemented in hardware, and the other components of the algorithm were implemented in software. The schematic of HW–SW interfacing (after this partition) is presented in Figure 7.

AHIP is usually placed after the de-mosaicing, ”debayering” module—the interface with the image sensor. The recommended color space of AHIP is YUV, but its internal modules can be configured to manage other color spaces, as well. Besides the interface with the sensor, AHIP also includes a data interface with the memory. In the pre-view mode, AHIP can be configured to handle data at one clock per pixel (the clock rate of the sensor). The data processed by AHIP can be sent to the main SDRAM memory of a target system. AHIP can be configured to provide a streaming interface to other modules, if needed.

The typical workflow with AHIP is the following: “live” data from the sensor or from the memory are sent to AHIP (one clock per pixel). In the period of a frame, some AHIP modules update the statistics and other modules write two-dimensional type “maps” (of pixels or pixel transformations) in the system memory, which has the advantage of a minimum delay (basically, data output has the cadence of pixels input). After completing a frame, AHIP triggers a CPU interrupt—under normal circumstances, this notifies the availability of fresh data, but error signaling may also occur. The CPU serving routine reads the AHIP registers to identify the reason for the interrupt. The CPU decides on AHIP reconfiguration—modules off or on—and, in this latter case, calculates their parameters. Finally, the CPU (before re-enabling the interrupts) is acknowledging its other threads, and the data from the HW accelerator became available (for the algorithms to start using them).

In this way, based on the pre-processing maps read from the system memory, the artificial intelligence predictor algorithm (implemented using neural networks—NN—and residing in the software part run by the CPU) manages to configure the AHIP functionality in the chosen mode and computes the AHIP parameters in an adaptive way. The “interrupts” principle is used in a modern “events processing” way—each complex signaling is accomplished as a directive plus parameters—”instance data” and ”support data”).

AI for hardware configuration is the software implementation part that dynamically decides what hardware modules are to be activated at a particular image scenario and computes the proper parameters (only) for these AHIP modules. The artificial intelligence has as input the pre-processing maps, and the output represents each AHIP sub-module “enable” decision and needed parameter values.

The orientations or conditions of the image capture—taken into consideration for an adaptive AI decision—may include: the direction of illumination of the object, object rotation in a plane, a variation in its 3D (three-dimensional) position, face colors, face shapes, face shading, face occlusion, how blurry the face is, defects in the way the eye is captured, whether the smile appears and to what extent, whether the eyes blink and to what extent, whether the mouth is open and how much—or a combinations of these features. Based on such criteria, the object’s data are accepted by one or more of the AI classifiers. The image can be one of a series of image streams that includes the object. Two or more classifiers can be applied if the object could not be detected by the first classifier(s). One or more tracking modules can be used to track, in consecutive images, any of these objects that have been detected.

Examples:

- In case an unevenly illuminated face appears, the artificial intelligence runs several classifiers in parallel on the acquired data, in order to detect different lighting conditions—the classifier designated as the most suitable also computes the relevant lighting conditions and a specific correction to be applied so that the face acquired obtains an uniform illumination; the artificial intelligence can optionally further consider the unevenly illuminated face (as it is) to be tracked, edited, memorized, or processed accordingly.
- In case lower light frames are observed in the input stream, the AI for configuration rewrites the LUT modules (inside AHIP) that provide the luminosity parameters for the Pixel PM blocks—see Figure 6.
- In case there are not enough details in the input frames, the AI for hardware configuration concludes that the objects presented are very big, so the image scaling parameters are set for a high down-scaling factor for the Pixel PM block in Figure 6.
- For a regular image exposure, the image data analyzed show enough information to be extracted by the ROI detector, so, in this case, artificial intelligence decides that most of the Pixel PM blocks are disabled (powered down—“off”).
- In case perspective corrections are needed (when the ROI is deformed due to position outside the image plane), the AHIP computes the vertical and horizontal alignment characteristics as frame PM. The perspective parameters are computed by artificial intelligence for instantiation and are used for the ROI distortion correction module inside AHIP.
- In the case of the ROI rotation in the plane, the region PMs are used to correct the rotation to a pre-defined angle. AI for instantiation determines the rotation angle for ROI and sets the proper rotation parameters per each ROI. In case the ROI does not need in-plane rotation, the region PMs are shut-down.

The final part of the software algorithm consists in an object detection AI that reads the ROI candidates generated by the hardware and applies an additional classifier for false positives rejection. The final list of objects, after the software filtering, is then written to memory as Final List of Detection.

## 4. Results

After the design of the hardware cores, and the implementation of the software components, the system was prototyped on an FPGA platform.

### 4.1. FPGA-Based Proof of Concept

**Figure 8:**
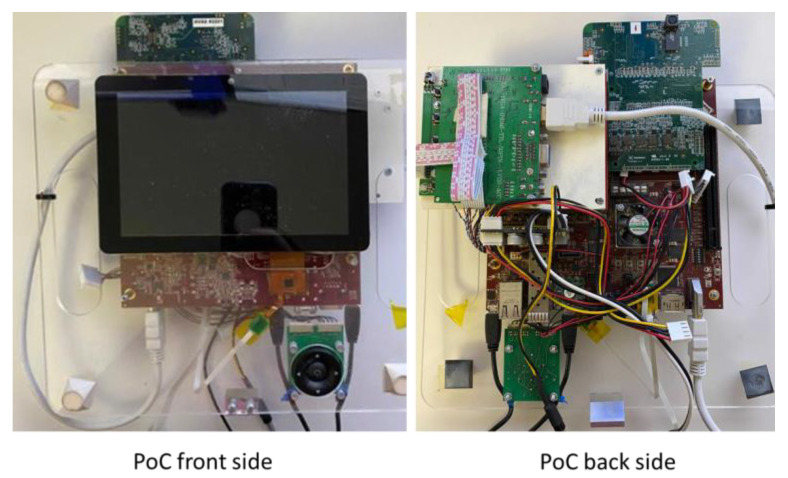
FPGA-based Proof of Concept (PoC) for objection detection.

Xilinx’s mini-ITX platform was preferred because the Zynq 7000 family FPGA device is powerful enough to implement the designed IP Cores and, in addition, has a fairly capable software sub-system, based on the ARM Cortex A9 microprocessor, which can run at 800 MHz. DRAM memory available on FPGA is quite generous (1 GB PS DDR3 SDRAM), and the available external interfaces are numerous and relatively easy to use—QSPI (Queued Serial Peripheral Interface), SATA-III (Serial AT Attachment, 3rd revision), microSD (micro- Secure Digital), Ethernet, HDMI (High-Definition Multimedia Interface), Audio input and output, USB (Universal Serial Bus), PCIe (Peripheral Component Interconnect Express), FMC (FPGA Mezzanine Card), HPC (High Pin Count), JTAG (standardized by the Joint Test Action Group), and GPIO (General-Purpose Input-Output). To demonstrate the full operation of the application, the peripherals for the acquisition and display of images were incorporated into a demonstrator, as shown in Figure 8. On the front of the FPGA platform, the image sensor card and the HDMI driver for the display were mounted. On the back side, a display was mounted for viewing the images and additional information, as well as a PCB (Printed Circuit Board) for simple control with buttons. All these components were mounted on a plexiglass support with handles for easy maneuverability.

To assess the quality of the application, analyses were performed on public reference databases, which allow comparing the implemented solution with other computer vision solutions presented in the literature, as well as with some commercial solutions. The object classes analyzed were persons and human faces.

### 4.2. Face Detection Testing

**Figure 9:**
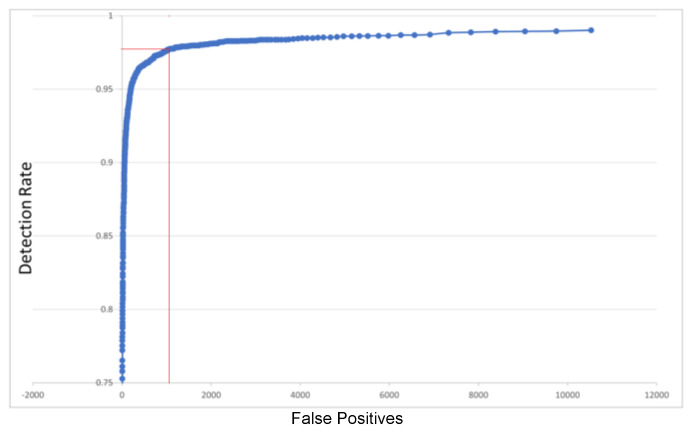
Face Detection Quality on FDDB Database.

For face detection, there are several public databases, but the most used is FDDB (Face Detection Data set and Benchmark) [43]. On this database, the results of our implementation of object detection are shown in Figure 9. It can be seen that the detection rate increases to over 97.5% for 1000 false positives (as the abscise marked in red on the plot of Figure 9). These results were validated versus the 30 detectors compared in a recent assessment [44], as seen in Table 1:

This assessment validated the correct operation of the ROI detector modules that take over the acquired frames and analyze them. To improve the quality of detections, the regions detected by this module (“Candidate Regions”) are further analyzed in the software (Object Detection algorithm) for the removal of any mis-detected regions (the “false positives”). Appendix B provides multiple samples of face detection performed on the FDDB dataset.

### 4.3. Person Detection Testing

**Figure 10:**
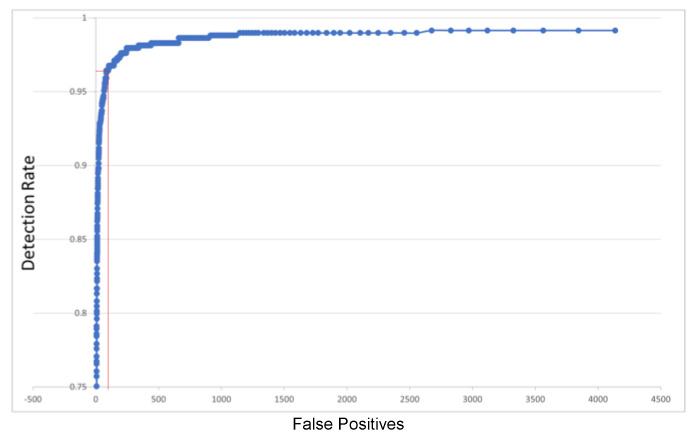
Person Detection Quality on INRIA Database.

The same assessment, on a public database for the persons detection, is presented in Figure 10. In this case, the database from INRIA (Institut National de Recherche en Informatique et Automatique) [45] was used, a well known reference for person detection. The results were validated versus the detectors proposed in [46,47]. The NN-specific detection rates are better than those that can be obtained with traditional SVM detectors that extend the HOG model of [45] with HikSVM (Histogram intersection kernel SVM—trained on the INRIA dataset).

We have unified the ordinate of both detection quality plots in Figure 9 and Figure 10, using the DR (Detection Rate). This is simple and relevant, as the Miss Rate (more common in person detection assessment) is MR = 1−DR (see Appendix C for details on qualitative and quantitative measures for detection). Regarding the abscise (of common person detection assessment plots), this is frequently FPPI (False Positives Per Image)—then a simple division of the unified abscise False Positives (FP) by the total number of images. Thus, the unified DR(FP) plots can be easily compared with MR(FPPI) or other curves, for any discussion on performance. For instance, in [48], it is shown that FPPW (FPPWindow) “could not reasonably evaluate the merits” of the (person-) pedestrian detection algorithms, so FPPI is preferred.

As in [46,47], many references on pedestrian detection focus on 0.1 as the central FPPI for representative MR relevant in comparative performance assessment. As the total number of images in INRIA is 902, FPPI = 0.1 is corresponding, in our case, with FP = 90.2 (as the abscise marked in red on the plot of Figure 10), for which our DR = 0.96532, then our representative MR = 3.468%, a good value according to Table 2.

### 4.4. Object Detection for Different Orientations

**Figure 11:**
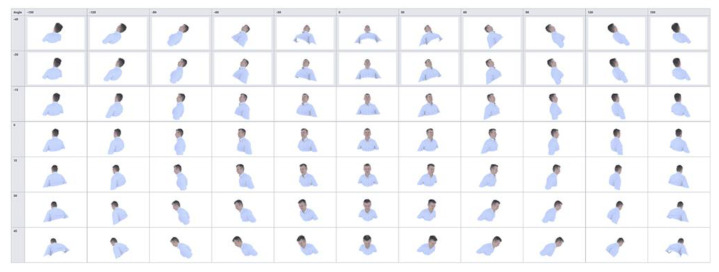
Orientation examples for face detection database.

**Figure 12:**
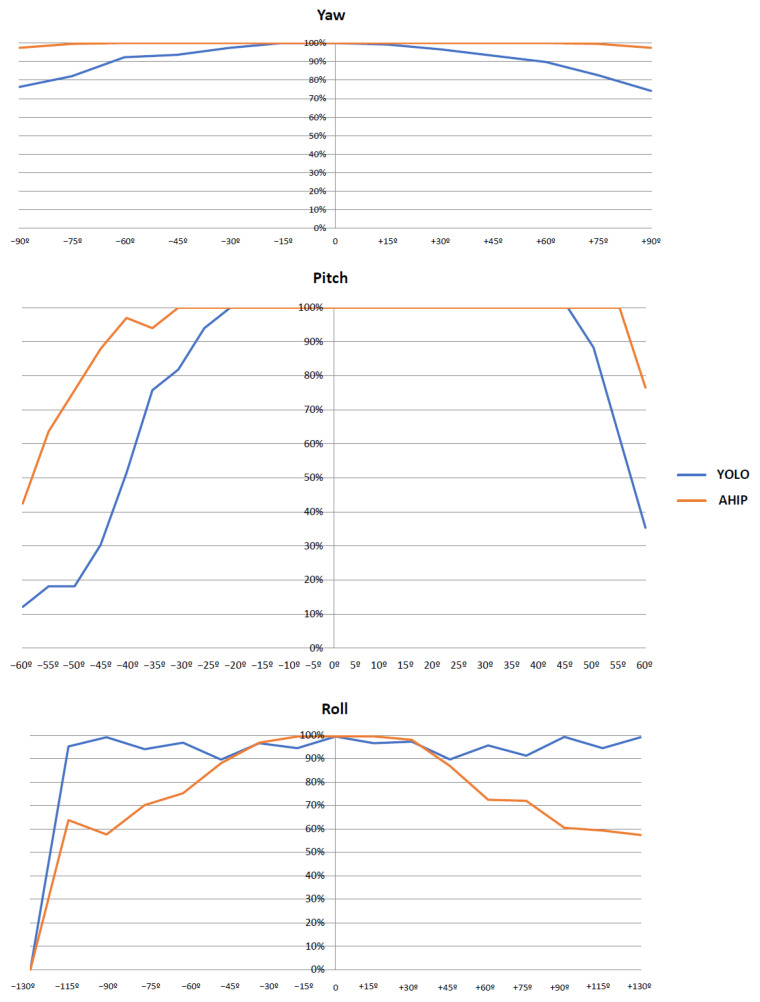
Face Detection Results Based on Orientation.

Often, these statistics are not enough. Object detection must work correctly for different orientations of the object. For this purpose, we created three-dimensional models of the objects, and the detection results for each orientation were then analyzed. Figure 11 shows a three-dimensional model oriented at different angles of rotation (outside the plane of the image) on both horizontal and vertical axis, and Figure 12 presents the results obtained for the 2 angles of rotation outside the plane of the image (to the left or to the right, around the horizontal axis front-rear—”Roll”/up or down, around the left-right horizontal axis—”Pitch”), as well as in the plane of the image (trigonometric or inverse-trigonometric, around the vertical axis—”Yaw”).

In this case, two different solutions were assessed, a public one, based on the YOLOv4 NN (in blue) and the implementation of our solution in hardware, using AHIP (in orange).

## 5. Conclusions

In the relationship between artificial intelligence and computer vision hardware, we have shown how AI-assisted decisions are made over the configuration of the hardware computer vision system. For this purpose, we have defined and implemented a hardware acceleration sub-system for image processing, AHIP (Advanced Hardware for Image Processing) that illustrates the concept of adaptive resource integration. This sub-system works as a collection of hardware modules that implement primitives for image processing. The control of the sub-system is done by the artificial intelligence algorithms that decide which hardware blocks are used and in what way. Artificial intelligence decides whether certain blocks should be selected (actually used) or not (placed in the active backup state and bypassed by the data flow). Therefore, additional parallel processing blocks can be included in the hardware architecture, being put into operation only when the artificial intelligence algorithm decides to do so.

The basic computing hardware modules interconnect to create feature maps—higher-level processing primitives. To optimize memory access, multiple calculation blocks of primitives can be coupled to read the data from the memory only *once.* Operation in a systolic matrix system—typical in image processing—assumes that each hardware module has very low latency, and thus the chained working mode allows for very fast calculation of primitives. As a result, image primitives are available shortly (a few CLK steps) after receiving the image. As a result, pipelining is possible—during a frame, its image primitives are calculated and, at the same time, the high-level image processing algorithm is run on the previous frame. In addition, AHIP allows the combination of primitives from successive frames to create higher-level primitives that are more efficient in terms of size and usage.

An original contribution is the anticipative feature of real-time processing: the artificial intelligence algorithm can predict details of the content of the current frame based on the analyzed information from the previous frames, and, in this way, the hardware blocks necessary for the optimal implementation of the image processing functions can be selected. The processing chain is thus dynamically and anticipatively re-configured. Additionally, each hardware block can be parameterized by the AI control algorithm. To increase the precision of these anticipative decisions of the artificial intelligence, AHIP allows for the pre-definition of dimensions in hardware for the approximate ROI, which are then refined into software. ROI can be defined by an image analysis module. The data resulting from the calculation in hardware can be sent directly to the system memory or compressed adaptively—as the compression can have, as input, the information provided by the artificial intelligence, such as the ROI position.

Adaptive integration of hardware resources using AHIP has been demonstrated in an object detection use case. These original solutions proposed were validated in the implementation on FPGA of a successive series of demonstrators for object detectors. These systems operate in real-time, even at the lower frequencies supported by FPGA devices, which illustrates the great technological advantages of the solutions. The proof of improving the overall quality of object detection was an analysis performed on public reference databases for face and person detection, using the demonstrator built around the Xilinx Zynq 7000 FPGA. We obtained improved detection rates and also succeeded in exceeding the quality levels of YOLOv4 object detection. Due to dynamic parameter optimization, the performance, as shown in Figure 12, is better for out-of-the-plane pitch and yaw rotations of the object (and for the main domain of roll angles).

Regarding the computing time KPI, while the YOLOv4 is designed to run on GPU and high-performance systems (achieving 97 fps on devices as NVIDIA GTX 1080 Ti), the tiny-YOLO [36] can reach real-time operation (30 fps or higher) on devices, such as NVIDIA [10] Xavier AGX, Xavier NX, Jetson TX2, or Jetson NANO. The proposed method implemented on FPGA can obtain 30 fps while maintaining the quality of the algorithm.

The paper focuses on hardware–software partitioning of algorithms with dynamic parameter optimization. The illustration of practical solutions for proof-of-concept implementations was done in the domain of real-time object detection, but, nevertheless, this proposed solution could work also on other image processing applications, e.g., face recognition or object classification.

## Figures and Tables

**Table 1:** AHIP-based face detector quality, compared to the best five out of thirty reference detectors.

Face Detector	Detection Rate *
ACETRON	0.9752
AHIP	0.9750
FlashNet	0.9733
LFFD (Light and Fast Face Detector)	0.9731
RetinaFace-mobile	0.9725
FCPU (Face-CPU)	0.9700

* for 1000 false positives.

**Table 2:** AHIP-based pedestrian detector, compared to the eight newest and traditional (*) detectors.

Pedestrian Detector	Miss Rate
AHIP	3.468%
Saeidi-Arabsorkhi [46]	6.18%
PCN (Part and Context Network) [46]	6.86%
F-DNN (Fused DNN) [46]	6.78%
YOLOv2 * [47]	11.29%
SketchTokens * [46,47]	13.32%
HikSVM* (Histogram Intersection Kernel) [47]	42.82%
HOG * [46,47]	45.98%
VJ * (Viola-Jones) [46,47]	72.48%

**Figure A1:**
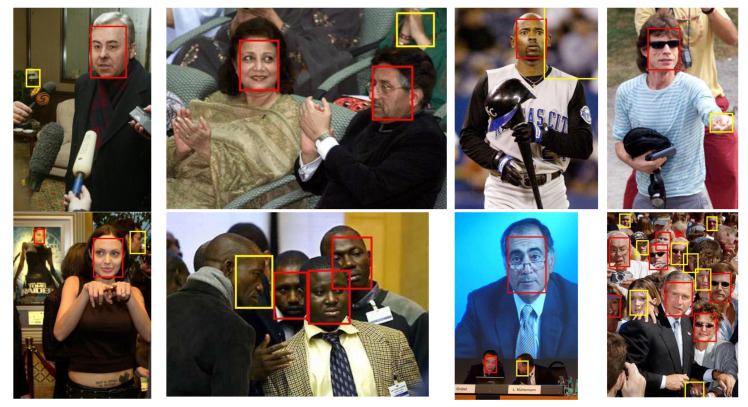

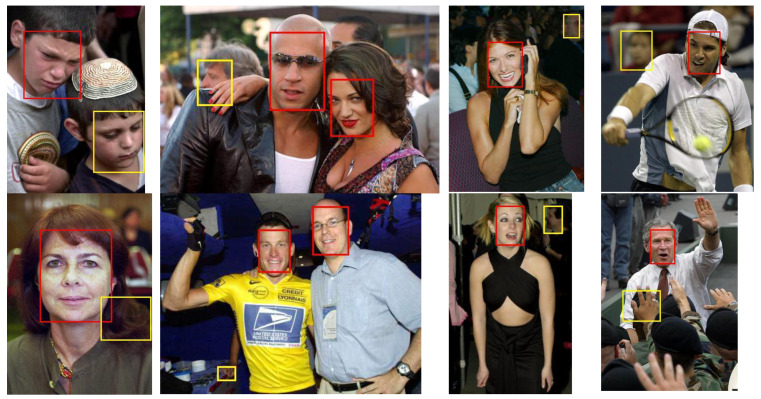
Samples of face detection on FDDB, with colored rectangles for labelling performed during the testing.

**Figure A2:**
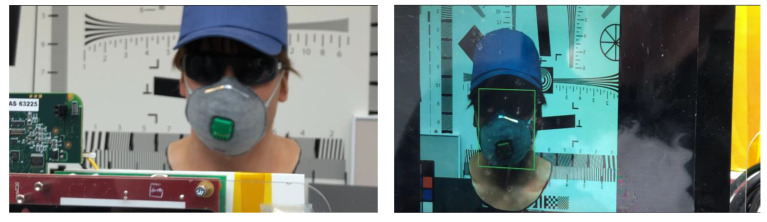
RT operation of the FPGA-based ”PoC”—the masked subject and the camera system board (**left**); the result of detection (**right**).

**Figure A3:**
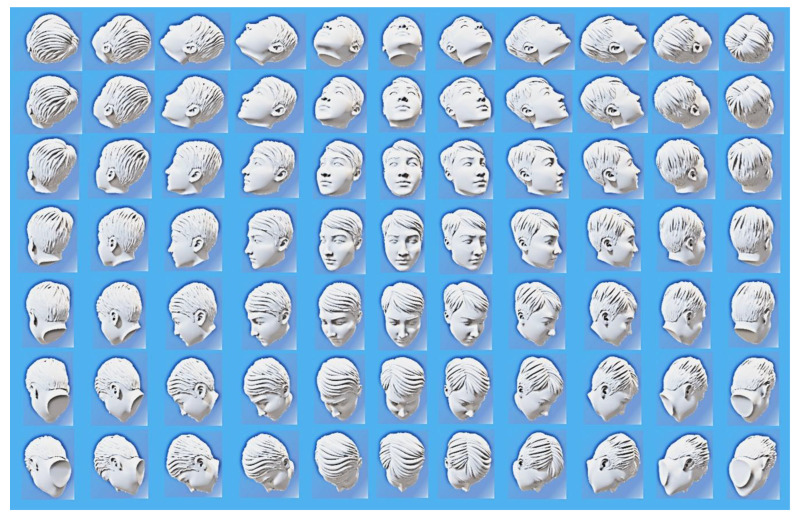
Three-dimensional Models generated in MS Office—with a step of 30° for yw and 20° for pitch.

## Data Availability

Data sharing is not applicable to this article.

## Appendix A

This appendix gives details on the quality of detection. The quality of algorithms has different aspects, depending on their type and destination. In the context of our solutions, quality is considered in terms of correctness and relevance (efficiency is conditioned more by KPIs, such as processing capacity or optimized memory usage).

An important category of CV algorithms is based on classification. For this category of algorithms, there are two common plots of quality: the P–R (Precision–Recall) curve and the ROC (Receiver Operating Characteristic) curve [49]. These two plots are done by varying one parameter—for example the detection threshold—and running multiple iterations of the algorithm for each parameter value (e.g., the number of runs can be at least one order of magnitude greater than the inverse of the accepted calculations’ tolerance).

Both common ways of expressing the quality of algorithms take into account the following values, related to a certain number of measurements–samplings–observations that, in the CV context, are detection attempts that can declare–indicate (correctly or incorrectly) the existence of a targeted object or its non-existence (correctly when it is missing, or incorrectly when it is present, but its absence is declared):

- *TP* (“True Positives”)—the number of existing objects detected (the algorithm response is correct)
- *TN* (“True Negatives”)—the number of non-existent objects declared as undetected (the algorithm response is correct)
- *FP* (“False Positives”)—the number of non-existent objects declared as detected (the algorithm response is incorrect)
- *FN* (“False Negatives”)—the number of existing undetected objects (the algorithm response is incorrect).

Based on this limited number of measurements, the following measures can be defined:

Precision (correct detections from all declared detections)

(A1) $$P=\frac{TP}{TP+FP}$$

and Recall (correct detections out of all possible detections in the ideal case, on that set of measurements)

(A2) $$R=\frac{TP}{TP+FN}$$

As *TP*, *TN*, *FP*, and *FR* values may vary in the case of the classification algorithm discussed (in general, with the modification of the respective parameter chosen—e.g., detection threshold), “quality scores”, such as harmonic mean, can be calculated. The “*F*_1_ score” is a good benchmark for the performance of the algorithm:

(A3) $$F_{1}=2\frac{P\cdot R}{P+R}$$

Another metric (more relevant, but less frequently used in practice) could be the area below the *P*(*R*) curve—AUC (Area Under the Curve), the parameter being the detection threshold. Similar to the *F*_1_ score, AUC can also be adopted as the single benchmark for the quality of the classification algorithm.

It is sometimes more intuitive to use another curve to render the quality of the classification algorithms, namely, the ROC. This takes into account the *TPR* (True Positive Rate) and *FPR* (False Positive Rate) metrics that are calculated as follows:

(A4) $$TPR=\frac{TP}{TP+FN}$$

(A5) $$FPR=\frac{FP}{FP+TN}$$

It is noticed that *TPR* is the precision, *P,* and that *FPR* indicates a dual mode, relative to the total cases of missing (real non-existence) of the object, as well as how many times it was detected as present (incorrectly declared as existing).

The ROC curve represents *TPR*(*FPR*) with the above-mentioned parameter and can also be relevant for the quality of the algorithm.

*TPR* (=*P*) is also called the hit rate or the detection rate (*DR*).

The miss rate (*MR*) is also very used, being also called FNR (False Negative Rate):

(A6) $$FNR=\frac{FN}{TP+FN}\;\;\left(=MR=1-DR\right)$$

For multiple classes of objects, the mean value of Precision, mAP (mean Average Precision)*,* can be considered. This is calculated as the average of the precision value for all classes of objects in a database. This benchmark is often used for public databases, such as MsCoco (Microsoft Common Objects in Context) [50].

These benchmarks for the quality of algorithms are useful strictly for classification problems. Usually, in the case of object detection, it is necessary not only to correctly classify the object, but also to find its exact position in the image. Therefore, detections are considered correct only if the “Jaccard index” IoU (Intersection over Union) is above a certain threshold —the area of the intersection relative to the area of the union of the determined “boxes” (contours of the determined framing-location) [51].

## Appendix B

This appendix provides multiple samples of face detection on the FDDB dataset—Figure A1. They were used for algorithm validation.

The red rectangles are drawn around correctly labeled faces (corresponding to the ground-truth), while the yellow rectangles are labels for false positives. Please note that the false positives are caused by poor focus or extreme yaw/pitch angles.

Figure A2 shows the real-time operation of the FPGA-based ”PoC” (Proof of Concept) of the proposed hardware–software system—one can notice the display and the camera system board depicted in Figure 8.

## Appendix C

This appendix describes the object detection samples in detail. In order to comply with the research and publications ethics, regarding the involvement of human subjects, Figure A3 illustrates how a set of three-dimensional models can be generated in MS Office.

Nevertheless, for testing the proposed solution, real photos of the 1st author were used, and they were shot in different positions (Figure 11) by the 2nd and 3rd author.
